# Kinetics and Determining Factors of the Virologic Response to Antiretrovirals during Pregnancy

**DOI:** 10.1155/2009/621780

**Published:** 2010-01-10

**Authors:** Adriana Weinberg, Jeri E. F. Harwood, Elizabeth J. McFarland, Jennifer Pappas, Jill Davies, Kay Kinzie, Emily Barr, Suzanne Paul, Carol Salbenblatt, Elizabeth Soda, Anna Vazquez, Charles A. Peloquin, Myron J. Levin

**Affiliations:** ^1^Department of Pediatrics, University of Colorado Denver School of Medicine, Aurora, CO 80045, USA; ^2^Department of Pediatrics, The Children's Hospital, Aurora, CO 80045, USA; ^3^Department of Obstetrics and Gynecology, University of Colorado Denver School of Medicine, Aurora, CO 80045, USA; ^4^University of Colorado Denver School of Medicine, Aurora, CO 80045, USA; ^5^Department of Infectious Diseases, National Jewish Hospital, Denver, CO 80206, USA

## Abstract

HIV-infected pregnant women with undetectable plasma HIV RNA concentrations at delivery pose a minimal risk of vertical transmission. We studied the kinetics and the determinants of the virologic response to antiretroviral therapy in 117 consecutive pregnancies. Patients who initiated therapy during pregnancy had a VL decrease of 2 and 2.5 log_10_ after 4 and 24 weeks, respectively. Therapeutic drug monitoring (TDM) of the protease inhibitors administered in doses recommended for nonpregnant adults resulted in below-target concentrations in 29%, 35%, and 44% of 1st, 2nd, and 3rd trimester measurements, respectively, but low drug concentrations did not correlate with virologic failure. Demographic characteristics, antiretroviral experience prior to pregnancy, baseline VL, or use of specific antiretrovirals did not affect the virologic response. Adherence to ≥95% of prescribed doses and utilization of psychosocial services were associated with undetectable plasma HIV RNA at delivery. In conclusion, the virologic responses of pregnant and nonpregnant adults share similar charactersitics.

## 1. Introduction

Highly active antiretroviral (ARV) therapy (HAART) has decreased HIV mother-to-child transmission (MTCT) to <2% in the US and other countries where ARVs are readily available [1–5]. This was achieved by suppressing maternal HIV replication. In addition, scheduled Cesarean section decreases the risk of MTCT [1, 6, 7]. Recent studies, however, suggest that Cesarean sections are not of significant benefit when plasma HIV RNA is undetectable at delivery [2, 3]. Since Cesarean sections increase the cost of deliveries and risk of maternal complications, understanding the kinetics of the virologic response to HAART during pregnancy may avoid performing unnecessary Cesarean sections.

The dominant risk factor for MTCT is high maternal viral load at delivery [8]. In nonpregnant individuals, HAART generally decreases HIV plasma RNA and increases CD4+ cells in parallel. This relationship has prompted the use of CD4+ counts as a surrogate measure of response to therapy when plasma HIV RNA measurements are not readily available. In pregnancy, however, this approach may not be valid, as pregnancy is associated with lower CD4+ numbers. The kinetics of plasma HIV RNA and CD4+ cells in response to HAART during pregnancy have been reported in separate studies [9, 10]. The information provided by these studies suggested that there may be a disconnect between changes in CD4+ cells and plasma HIV RNA during pregnancy. In this study, we analyzed these two parameters in parallel. 

Another important issue for prevention of MTCT (PMTCT) is optimization of drug regimens during pregnancy, which typically include 2 nucleoside reverse transcriptase inhibitors (NRTI) and a protease inhibitor (PI). Pharmacokinetics and therapeutic drug monitoring (TDM) studies during pregnancy showed that absorption and distribution of PIs differ in 3rd trimester pregnant women compared with nonpregnant adults, such that dose adjustments may be warranted [11–13]. However, there is a lack of information regarding PI pharmacokinetics during early pregnancy. 

To address these issues, we conducted a retrospective chart review of pregnancies managed by the Children's Human Immunodeficiency Program (CHIP) in Denver. In August 1997, CHIP became the reference center for the care of HIV-infected pregnant women in Colorado and neighboring states. These women come from a relatively broad patient population that is highly representative of the HIV epidemic in the Southwestern US.

## 2. Patients and Methods

### 2.1. CHIP PMTCT Program

Patients were cared for by a multidisciplinary team, comprising specialists in adult and pediatric infectious diseases, obstetrics and maternal-fetal medicine, nursing, social work, mental health, and nutrition. Basic HIV-specific treatment consisted of ≥3 ARV including ≥2 classes of ARV. HIV genotype was determined before treatment initiation in patients with plasma HIV RNA ≥1000 copies/mL. Adherence was facilitated by distributing pill boxes, watches, and pagers, office counseling or home visits, and direct observed therapy. Plasma HIV RNA and CD4+ cells were measured at 2–6 week intervals. 

PI plasma concentrations were assessed using high-pressure liquid chromatography assays performed at the National Jewish Hospital pharmacology laboratory, certified by the AIDS Clinical Trials Group and compliant with the Clinical Laboratory Improvements Amendments. For TDM, drugs were administered in clinic with or without food, as indicated. Trough concentrations were obtained immediately before the dose. Peak concentrations of lopinavir (LPV) were measured 4 hours after administration of LPV/ritonavir (LPVr) and of other PIs 2 hours after administration of the drug.

Treatment was modified based on drug levels, virologic response, safety, and tolerability. Centralized treatment decisions were made by the physician in charge of the maternal care of the PMTCT program in consultation with the primary infectious diseases provider of the patient, if different from the PMTCT physician.

The HIV-specific obstetrical plan included intravenous zidovudine (AZT) administration during delivery except for patients on stavudine, who continued their oral medications. Cesarean sections were scheduled at 38 weeks of gestation for consenting patients with VL > 1000 copies/mL at ≥34 weeks gestation, as indicated by other medical obstetrical conditions or patients' choice.

Infants were treated during the first 6 weeks of life with AZT. When the maternal VL at the last visit prior to delivery was ≥5,000 copies/mL or >1000 copies/mL with vaginal delivery, the neonate received 2 additional drugs from 2 ARV classes for the first 4 weeks of life.

### 2.2. Chart Review

This study was reviewed by the local IRB and deemed exempt from IRB approval due to the retrospective nature of the study and absence of disclosure of any personal information. Pregnancies with delivery between Aug. 1997 (inception of the CHIP PMTCT program) and Dec. 2005 (initiation of chart review) were identified in the CHIP database. Information was abstracted for pregnancies with ≥16-week duration and ≥2 visits at CHIP, using study-specific forms.

### 2.3. Statistical Analysis

Analyses assumed a two-sided test of hypothesis with a 0.05 significance level. Associations between categorical variables were determined using Fisher's exact test. Wilcoxon rank-sum test was used for comparisons of continuous variables between two groups. Changes in VL and CD4+ T-cell counts were tested using a signed-rank test. 

To determine the kinetics of the virologic response, log_10_ VL was modeled as smooth functions of time on treatment using a natural cubic B-spline transformation on time [14]. This analysis was run in the SAS mixed procedure (SAS Institute, Inc.) and the B-splines were calculated using the *ns*( ) command in Splus v8.0 (Insightful Corp). A random intercept was included in all models and a random slope was modeled using natural cubic B-splines. Akaike's Information Criterion was used to determine the final model [15]. We allowed a maximum of 5 and 3 degrees of freedom (df) for the fixed and random effect B-splines, respectively, and the final model included the maximum, resulting in the most flexible model. The same flexible analysis was used to model CD4+ T-cell counts over time. The final model included 5 and 1 df for the fixed and random effects, respectively. 

As VL was measured using different assays, the lower limit of detection varied. Six limits exist in our data: <20, 20–50, <50, <200, 200–400, and <400. In preparation for the B-spline analyses, we imputed plasma HIV RNA copies/mL such that those with (a) <20 were set to 10, (b) 20–50 to 35, (c) <50 to the mean of all values <50 (real and imputed), (d) <200 to the mean of all values below 200 (real and imputed), (e) 200–400 to 300, and (f) <400 to the mean of all values <400 (real and imputed). The log_10_ transformation was conducted after the imputation.

## 3. Results

### 3.1. Demographics and HIV Disease Characteristics of the Study Population

Medical records were available for 117 of 123 pregnancies that met inclusion criteria. These involved 12 women with 2 pregnancies (Table 1). The median age at delivery was 30 years. Thirty-one patients (30%) were black and 41 (39%) were Hispanic. A third of patients most likely acquired HIV in a foreign country. 

The most common risk factor for HIV acquisition was heterosexual intercourse, reported by 97 patients (92%). In 70 patients (67%), the diagnosis of HIV infection was established before pregnancy. Of the 35 patients diagnosed with HIV during pregnancy, 11 (34%) were diagnosed during the first trimester, 16 (50%) during the second trimester, and 5 (16%) during the third trimester. 

At the first pregnancy visit, the median VL was 2657 copies/mL including 78 (72%) with >400 copies/mL. The median CD4+ T-cells was 450 cells/*μ*L; 13 women (12%) had <200 cells/*μ*L. In 51 pregnancies (44%), the women were ARV naϊve and in 59 (50%) ARV experienced, including 29 patients who became pregnant on therapy.

### 3.2. Pregnancy Outcome

Median gestational age at delivery was 38 weeks (interquartile range (IQR) = 37–40 weeks). Thirty-six women (31%) delivered by scheduled Cesarean section. The 114 single and 3 twin pregnancies resulted in, 117 infants without HIV infection, 1 fetal demise, 1 stillbirth and 1 neonate who died of sepsis at 1 day of life. Of the 117 neonates who lived >1 day, 98 (84%) received AZT and 19 (16%) HAART. These infants were not infected with HIV. The incidence of MTCT was 0 with a 95% confidence interval = 0% to 3%.

### 3.3. ARV Utilization during Pregnancy

At delivery, 106/114 (94%) pregnant women were on HAART (≥3 drugs from ≥2 classes); 7 women (4%), who did not tolerate PIs or NNRTIs, were on 2 or 3 NRTIs; and 1 woman, who delivered shortly after referral precluding a change in therapy, was on AZT monotherapy. The most common drugs at delivery were AZT (*N* = 89), lamivudine (*N* = 108), stavudine (*N* = 22), nevirapine (NVP; *N* = 19), nelfinavir (NFV; *N* = 55), and LPVr (*N* = 25).

The median duration of continuous therapy up to delivery was 22 weeks (IQR of 15 to 35 weeks). Among 84 women who started ARV during pregnancy after being off therapy for several months or never being on therapy, the median duration of therapy during pregnancy was 20 weeks (IQR of 11 to 25 weeks). Of these, 77 (92%) received ARV for ≥4 weeks and 61 (73%) for ≥12 weeks.

### 3.4. Adherence

Adherence was measured by pill counts, pharmacy refill histories, MEMS caps, and detailed interviews in 44 pregnancies. Of these, 36 women (82%) took ≥95% doses of the prescribed medication, 7 (16%) took 50% doses–95%, and 1 took <50% doses. The analysis of adherence by ARV regimen (Table 2) did not reveal any differences across regimens.

### 3.5. Psychosocial Support

This was provided in 76 of 117 pregnancies. A median of 7 support interventions per pregnancy took place (IQR = 4 to 13). These included case management visits (*N* = 59), referrals to housing agencies (*N* = 31) and peer advocacy groups (*N* = 30), food (*N* = 59) and financial (*N* = 56) assistance, and psychoeducational group activities (*N* = 37).

### 3.6. Virologic Response to ARV during Pregnancy

For all study participants, the median VL at delivery was 51 copies/mL (IQR < 20 to 292; *N* = 95), which represented a significant decrease compared with the first pregnancy visit median of 2657 copies/mL (*P* < .0001). At the last visit before delivery, 80 women (82%) had plasma HIV RNA <400 copies/mL and 53 women (56%) had < 50 HIV RNA copies/mL. 

**Figure 1:**
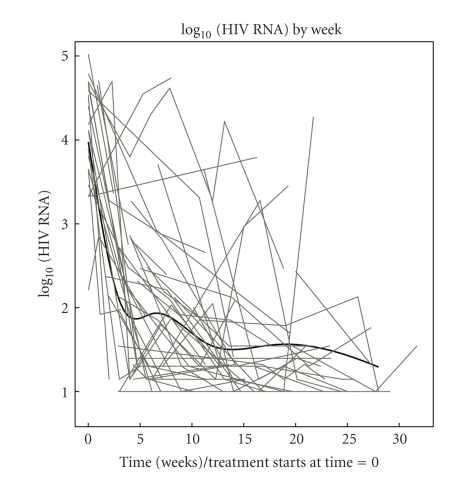
Kinetics of the plasma HIV RNA in pregnant women on HAART. Data were derived from 76 pregnant women who initiated or reinitiated HAART during pregnancy. 0 weeks indicates the beginning of therapy. Grey lines represent individual plasma HIV RNA trajectories. The black line represents the population curve generated by the mixed-model analysis. The predictions of the model were confirmed by additional analyses which showed that at 4 weeks of HAART, 42% of mothers had <100 HIV RNA copies/mL of plasma; at 12 weeks, 42% had <50 copies/mL; and at 24 weeks, 56% had <50 copies/mL. However, it should be noted that the model's estimated rise between 5 and 7 weeks is likely not real.

To determine the kinetics of the virologic response to HAART during pregnancy, we applied a mixed-model analysis to data from 76 of 84 patients who initiated or reinitiated HAART during pregnancy and had multiple tests during pregnancy (Figure 1). The model, which uses all available data to estimate the mean VL trajectory over time, estimated that after 4 weeks of HAART the log_10_ VL decreased by ~2 logs from baseline to a back-transformed mean <100 copies/mL. Both at 12 and 24 weeks of HAART, the estimated back-transformed mean VL was <50 copies/mL. We verified the predictions made by the model by calculating the proportions of subjects with VL < 100 copies/mL at 4 weeks and <50 copies/mL at 12 and 24 weeks of HAART. Of 39 women with VL measurements at 4 weeks of HAART, 15 (42%) had <100 copies/mL; at 12 weeks of HAART, 11 of 26 women (42%) had <50 copies/mL; and at 24 weeks of HAART, 9 of 16 women (56%) had <50 copies/mL. These data confirmed the predictions of the model.

### 3.7. Immunologic Response to ARV during Pregnancy

At delivery, the median CD4+ T-cell number was 540 cells/*μ*L (IQR of 416 to 678; *N* = 92): 92% of values >200 cells/*μ*L. The median CD4+ T-cell increase between first pregnancy visit and delivery was 88 cells/*μ*L (*P* < .0001 by signed-rank test; *N* = 92). 

**Figure 2:**
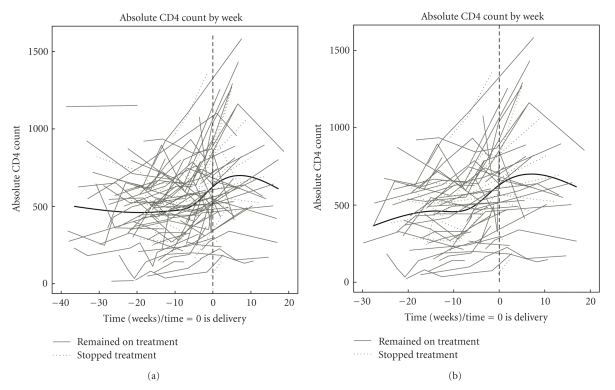
Kinetics of CD4+ counts in HIV-infected pregnant women on combination ARV. (a) Data were derived from 100 pregnant with available data who started therapy before or during pregnancy. (b) Data were derived from 76 pregnant women who started therapy during pregnancy after being off therapy for several months or never being on therapy. Time 0 indicates delivery. Grey lines represent individual CD4+ trajectories. The black line represents the population curve generated by the mixed-model analysis.

In the mixed-model analysis used to elucidate the kinetics of CD4+ T-cells during pregnancy, we set the reference time at delivery to take into account the upward effect of ARV and the downward effect of pregnancy on the CD4+ T-cells. The model indicated that the CD4+ T-cells started to increase at 6 weeks prior to delivery and peaked at approximately 8 weeks after delivery in all subjects (Figure 2(a)) and in those who initiated or reinitiated HAART during pregnancy (Figure 2(b)). Among 54 women with postpartum data, there were no appreciable differences in postpartum CD4+ T-cell numbers of 36 women who continued therapy for ≥12 weeks, compared with those of 18 women who discontinued therapy immediately after delivery. An additional mixed-model analysis allowed for separate CD4+ T-cell trajectories postdelivery for the 18 women who discontinued therapy immediately after delivery and the 36 women who continued therapy for ≥12 weeks. From this model, the estimated CD4+ T-cell count at 12 weeks postpartum was 763 and 679, for women who stopped therapy and continued therapy for ≥12 weeks, respectively, a difference of 84 (*P* = .29).

### 3.8. PI Plasma Concentrations during Pregnancy

Thirty-two pregnant women had ≥1 PI TDM for a total of 92 measurements. TDM was performed at a median of 5.5 weeks after initiation or change in therapy (IQR of 3.5 to 30 weeks). Eighty-two TDM assays were performed in women taking PI doses recommended for nonpregnant adults (regular) and 10 in women taking increased doses. 

Table 3 depicts the TDM results from women on regular PI doses. There were 7 measurements in the 1st trimester. Of these, 4 (57%) were within the targeted range (normal), 2 (29%) were low, and 1 (14%) was high. The 57 TDM assays from the second trimester included 34 (60%) normal, 20 (35%) low, and 3 (5%) high results. The 18 TDM assays from the 3rd trimester included 9 (50%) normal, 8 (44%) low, and 1 (6%) high results. Across all gestational ages, 57% of the TDM results were normal, 37% low and 6% high. The distribution of TDM results did not significantly differ across trimesters (Fisher's exact test, *P* = .70).

The analysis of the plasma concentrations achieved by the most commonly used PI in this study, LPVr, NFV, and saquinavir (SAQr), showed that 75% of all LPV measurements were normal and 25% were low across all trimesters; 20% to 59% of the NFV levels were normal with a trend of decreasing concentrations from 1st to 3rd trimester; and 30 to 50% of SAQ concentrations were normal. 

There were 10 PI TDM results during the 2nd or 3rd trimester in women who received increased PI doses (6 received LPVr 533/133 mg bid and 3 NFV 1500 mg bid). Of these, 3 measurements (30%) were below target.

### 3.9. Factors That Affected Virologic Response to Therapy during Pregnancy

To evaluate potential differences between HAART regimens with respect to VL decay during pregnancy, the mixed-model analysis of log_10_ VL described above (Virologic response to therapy) was used to compare NFV- (*N* = 49) versus LPVr-containing HAART (*N* = 20). Differences in estimated log_10_ VL between the regimens, calculated as NFV estimate minus LPVr estimate, were 0.01 (*P* = .96), 0.44 (*P* = .10), 0.39 (*P* = .12), and 0.09 (*P* = .78) at 4, 8, 12, and 24 weeks on treatment, respectively.

An analysis of the factors that could potentially affect the response to therapy defined by undetectable VL at delivery, including race, ethnicity, country of origin (as a surrogate of HIV-1 clade), magnitude of VL before initiation of therapy, ARV experience, HIV drug resistance (data presented elsewhere) [16], adherence, TDM, and utilization of psychosocial support services, was performed (Table 4). The data revealed significant associations only for adherence (*P* = .006) and psychosocial support (*P* = .010).

## 4. Discussion

To determine what length of HAART administration during pregnancy was needed to achieve undetectable VL, we analyzed the kinetics of the virologic response in women who started HAART during pregnancy. The time to undetectable VL did not differ from that previously reported in HIV-infected nonpregnant adults [17, 18], with a majority of pregnant women achieving VL < 50 copies/mL after 12 to 24 weeks of therapy. The corollary of this observation is that pregnant women who start HAART during or before the 2nd trimester are likely to achieve VL < 50 copies/mL at delivery. In contrast, women starting HAART less than 4 weeks before the anticipated date of delivery are likely candidates to scheduled Cesarean sections.

In our patient population, the rate of Cesarean section was similar to that reported for other areas in the US and lower than that reported in Europe, where the use of scheduled Cesarean section is twice as common as in the US [1, 19]. However, MTCT in this study was lower than that documented in contemporaneous studies in the US [4, 19] and similar to that reported in Europe [1, 3]. These differences may be explained by the fact that 94% of HIV-infected pregnant women in this study received HAART for PMTCT, which is a higher proportion than the rate of 50% to 70% documented in other studies [2–5]. Taken together, these observations suggest that high uptake of HAART may decrease the need of scheduled Cesarean sections for PMTCT [2, 3] without compromising protection of the neonate against HIV infection.

The analysis of the kinetics of CD4+ T-cells in pregnancy revealed a delay in the response to HAART compared with the kinetics of VL. In pregnancy, the most significant increases in CD4+ T-cell numbers occurred in the last 6 weeks of pregnancy and first 6 weeks of postpartum. This pattern was observed in women who started HAART before or during pregnancy and was independent of continuing HAART post-partum. This pattern is most likely due to physiologic changes of pregnancy, including the increased volume of distribution of white blood cells and/or hormonal changes depressing CD4+ T-cells. The implication of this observation is that a lack of increase of CD4+ T-cells in pregnant women may not indicate virologic failure.

An important question in the care of HIV-infected pregnant women is whether there is an ARV regimen more likely than others to rapidly decrease the VL in women diagnosed with HIV late in pregnancy. We did not find any significant differences in the kfinetics of VL as a function of the PI used in HAART. Previous studies showed that nonpregnant HIV-infected adults on NFV-containing regimens were less likely to achieve or maintain viral suppression compared with individuals receiving LPVr-containing regimens [20]. However, during the limited duration of therapy for PMTCT, this difference may not be relevant. These data suggest that although HAART is critical in order to achieve a satisfactory virologic response during pregnancy, the choice of PI has secondary importance with respect to virologic response. Patel et al. showed that NVP-based regimens are associated with faster decay of VL compared with NFV-based regimens [10]. In this study, the number of women who intitiated NVP during pregnancy was too low for a formal analysis. Further studies using NNRTIs that do not carry the same high risk of serious adverse events as NVP are warranted to determine whether an NNRTI-based regimen may be advantageous when therapy is initiated late in pregnancy.

With respect to plasma concentrations achieved by various PIs during pregnancy, we found that LPVr levels were below target in 20% to 25% of pregnant women on adult recommended doses across all trimesters, whereas for another PI, 35% to 80% of the levels were below target. Others have shown that up to 72% of women on LPVr do not reach target concentrations of LPV during the 3rd trimester [11, 12, 21]. Acosta et al. found SAQ plasma levels uniformly above target [22] during pregnancy, whereas in our study 50% of subjects had levels below target. Our findings on NFV plasma concentrations are in accordance with those of Nellen et al. who also found that 51% of pregnant women did not reach target concentrations [23]. Differences in drug concentrations across studies may be accounted by technical differences, effects of race and ethnicity on drug metabolism, and compliance with therapy. A novel contribution of our study is to reveal that plasma PI concentrations are often below target not only in the last trimester of pregnancy but also earlier in pregnancy. This suggests that other factors, in addition to the increased volume of distribution of the drugs, may contribute to this effect. The practical implication of our findings is to underscore the importance of performing TDM or other pharmacokinetic studies during the 1st and 2nd trimesters of pregnancy and also after each dose modification, since 30% of the pregnant women on increased PI doses had plasma drug levels below target. 

The use of TDM did not significantly affect the virologic response to therapy in this study. Some studies in nonpregnant HIV-infected adults showed improved virologic response to PI-containing regimens in subjects whose doses were adjusted based on TDM results [24–26], but one study failed to show any benefit [27]. This last study had a period of followup of only 12 weeks. Likewise, it may be difficult to assess the benefit of TDM in pregnancy due to the limited duration of therapy. Nevertheless, it is reasonable to assume that it would be undesirable to administer drugs at subtherapeutic levels to control a virus that has the ability to quickly develop drug resistance mutations [28], particularly if ARV continues to be administered after pregnancy. Further studies are warranted to determine the effect of TDM on the development of resistance to ARV.

Among multiple factors that could potentially affect virologic response to therapy, suppression of viral replication at delivery was positively associated with adherence, confirming previous observations [29]. We did not find significant differences in adherence between patients receiving once daily versus twice daily regimens or PI-containing versus sparing regimens, which is a novel finding, perhaps specific of pregnancy and has important implications for the clinical practice. Overall, ≥95% adherence was recorded in 82% of the women in this study, which is consistent with other reports [30]. 

Utilization of psychosocial support services was significantly associated with virologic response to therapy during pregnancy, which makes intuitive sense, but has not been previously demonstrated in a formal analysis. Although the association between utilization of psychosocial services and virologic success may indicate that women who are committed to medical treatment for PMTCT are also likely to seek resources to improve other aspects of their lives, it may also indicate that support services facilitate adherence to therapy [30–32]. 

In summary, undetectable VL at delivery was associated with lack of vertical transmission of HIV in this group of women who predominantly delivered by vaginal route. Undetectable VL was achieved as soon as 4 weeks after initiation of HAART in 50% of the patients and at 12 weeks in the vast majority. Achieving undetectable VL at delivery did not depend on the specific PIs or NNRTIs included in the treatment regimen. Adherence, which was important to reach undetectable VL at delivery, did not vary with the drugs included in the treatment regimen, either. The pattern that emerges is that critical factors for PMTCT without high utilization of Cesarean sections are initiating therapy ≥12 weeks prior to delivery with a regimen that is well tolerated and conducive to ≥95% adherence.

## Figures and Tables

**Table 1:** Demographics and baseline characteristics of HIV infection.

Characteristic	*N* or Median (% or IQR)
Patients	105
One pregnancy with CHIP	93 (89)
Two pregnancies with CHIP	12 (11)
Maternal age at delivery	30 (26, 34)
Race	
White	72 (68.6)
Black	31 (29.5)
Other	2 (1.9)
Ethnicity	
Hispanic	41 (39)
Not Hispanic	64 (61)
Country of origin	
African country*	15 (14)
Mexico	17 (16)
USA	66 (63)
Other	4 (4)
Unknown	3 (3)
HIV risk factors^†^	
IV drug use	12 (11)
Heterosexual sex	97 (92)
Transfusion	5 (5)
HIV diagnosis	
Prior to first pregnancy at CHIP	70 (67)
During pregnancy	35 (33)
ARV experience	
Naive	51 (44)
Experienced on therapy at the onset of pregnancy	29 (25)
Experienced off therapy at the onset of pregnancy	30 (26)
Unknown	7 (5)
Plasma HIV RNA at first visit	109 pregnancies
Median (range)	2657 (225, 16700)
>400 copies/mL	78 (72)
CD4+ count at first visit	108 pregnancies
Median (range)	450 (269, 628)
<200 cells/*μ*L	13 (12%)

*Includes Ethiopia, Liberia, Nigeria, Kenya, and Somalia.

^†^Nine patients have more than one risk factor.

**Table 2:** Adherence to the last ARV regimen used during pregnancy.

ARV regimen	*N* with ≥95% adherence/*N* total (%)
Any regimen	36/44 (82)
≥3-drug with PI	25/28 (89)
≥3-drug with NNRTI	3/5 (60)
≥3-drug with PI and NNRTI	3/5 (60)
NRTI only	5/6 (83)

**Table 3:** Distribution of therapeutic drug monitoring results of protease inhibitors during pregnancy.

	All	LPVr	NFV	SAQr	IDVr	RTV
1st trimester	7	4	2	1	0	0
Low	2 (29)	1 (25)	1	0	0	0
Normal	4 (57)	3 (75)	1	0	0	0
High	1 (14)	0	0	1	0	0
2nd trimester	57	28	17	10	0	2
Low	20 (35)	7 (25)	6 (35)	5 (50)	0	2
Normal	34 (60)	21 (75)	10 (59)	3 (30)	0	0
High	3 (5)	0	1 (6)	2 (20)	0	0
3rd trimester	18	5	5	4	2	2
Low	8 (44)	1 (20)	4 (80)	2 (50)	1	0
Normal	9 (50)	4 (75)	1 (20)	2 (50)	0	2
High	1 (6)	0	0	0	1	0

Numbers (%) represent pregnant women taking PI doses recommended for adults.

Abbreviations: IDVr = indinavir/ritonavir; LPVr = lopinavir/ritonavir; NFV = nelfinavir; RTV = ritonavir; SAQr = saquinavir/ritonavir.

**Table 4:** Relationship between virologic response to ARV during pregnancy and selective factors that may affect this response.

Factor	*N* (%) undetectable	*N* (%) detectable	*P* value
Country of origin			.16
US	39 (80)	10 (20)	
Mexico	11 (79)	3 (21)	
African country	15 (100)	0	
HIV plasma RNA at first visit			
≥50,000 copies/mL	6 (75)	2 (25)	.63
<50,000 copies/mL	72 (83)	15 (17)	
Prepregnancy ARV			.27
Naive	33 (77)	10 (23)	
Experienced	40 (87)	6 (13)	
TDM			.38
Performed	53 (79)	14 (21)	
Not performed	25 (89)	3 (11)	
Adherence			.006
<50%	1 (100)	0	
50% to 95%	3 (50)	3 (50)	
>95%	29 (94)	2 (6)	
Psychosocial support			.01
Utilized	63 (89)	8 (11)	
Not utilized	15 (63)	9 (37)	
